# A Nationwide Study of GATA2 Deficiency in Norway—the Majority of Patients Have Undergone Allo-HSCT

**DOI:** 10.1007/s10875-021-01189-y

**Published:** 2021-12-10

**Authors:** Silje F. Jørgensen, Jochen Buechner, Anders E. Myhre, Eivind Galteland, Signe Spetalen, Mari Ann Kulseth, Hanne S. Sorte, Øystein L. Holla, Emma Lundman, Charlotte Alme, Ingvild Heier, Trond Flægstad, Yngvar Fløisand, Andreas Benneche, Børre Fevang, Pål Aukrust, Asbjørg Stray-Pedersen, Tobias Gedde-Dahl, Ingvild Nordøy

**Affiliations:** 1 Section of Clinical Immunology and Infectious Diseases, Department of Rheumatology, Dermatology and Infectious Diseases, Oslo University Hospital, Rikshospitalet, Oslo, Norway; 2 Research Institute of Internal Medicine, Division of Surgery, Inflammatory Diseases and Transplantation, Oslo University Hospital, Rikshospitalet, Oslo, Norway; 3 Department of Paediatric Haematology and Oncology, Division of Paediatric and Adolescent Medicine, Oslo University Hospital, Oslo, Norway; 4 Department of Haematology, Oslo University Hospital, Oslo, Norway; 5 Department of Pathology, Oslo University Hospital, Oslo, Norway; 6 Department of Medical Genetics, Oslo University Hospital, Oslo, Norway; 7 Department of Medical Genetics, Telemark Hospital, Skien, Norway; 8 Norwegian National Unit for Newborn Screening, Division of Paediatric and Adolescent Medicine, Oslo University Hospital, Oslo, Norway; 9 Institute of Clinical Medicine, University of Tromsø, Tromsø, Norway; 10 Department of Paediatrics, University Hospital of North Norway, Tromsø, Norway; 11 Department of Haematology, The Clatterbridge Cancer Centre NHS Foundation Trust, Liverpool, UK; 12 Centre for Cancer Cell Reprogramming, Institute for Cancer Research, Oslo University Hospital, Oslo, Norway; 13 Department of Medical Genetics, Haukeland University Hospital, Bergen, Norway; 14 Institute of Clinical Medicine, University of Oslo, Oslo, Norway; 15 Department of Paediatrics, Division of Paediatric and Adolescent Medicine, Oslo University Hospital, Oslo, Norway

**Keywords:** GATA2 deficiency, Hematopoietic stem cell transplantation, Germline mutation, Hematologic neoplasms, Primary immunodeficiency

## Abstract

**Purpose:**

GATA2 deficiency is a rare primary immunodeficiency that has become increasingly recognized due to improved molecular diagnostics and clinical awareness. The only cure for GATA2 deficiency is allogeneic hematopoietic stem cell transplantation (allo-HSCT). The inconsistency of genotype–phenotype correlations makes the decision regarding “who and when” to transplant challenging. Despite considerable morbidity and mortality, the reported proportion of patients with GATA2 deficiency that has undergone allo-HSCT is low (~ 35%). The purpose of this study was to explore if detailed clinical, genetic, and bone marrow characteristics could predict end-point outcome, i.e., death and allo-HSCT.

**Methods:**

All medical genetics departments in Norway were contacted to identify GATA2 deficient individuals. Clinical information, genetic variants, treatment, and outcome were subsequently retrieved from the patients’ medical records.

**Results:**

Between 2013 and 2020, we identified 10 index cases or probands, four additional symptomatic patients, and no asymptomatic patients with germline *GATA2* variants. These patients had a diverse clinical phenotype dominated by cytopenia (13/14), myeloid neoplasia (10/14), warts (8/14), and hearing loss (7/14). No valid genotype–phenotype correlations were found in our data set, and the phenotypes varied also within families. We found that 11/14 patients (79%), with known GATA2 deficiency, had already undergone allo-HSCT. In addition, one patient is awaiting allo-HSCT. The indications to perform allo-HSCT were myeloid neoplasia, disseminated viral infection, severe obliterating bronchiolitis, and/or HPV-associated in situ carcinoma. Two patients died, 8 months and 7 years after allo-HSCT, respectively.

**Conclusion:**

Our main conclusion is that the majority of patients with symptomatic GATA2 deficiency will need allo-HSCT, and a close surveillance of these patients is important to find the “optimal window” for allo-HSCT. We advocate a more offensive approach to allo-HSCT than previously described.

**Supplementary Information:**

The online version contains supplementary material available at 10.1007/s10875-021-01189-y.

## Introduction

GATA2 deficiency is a rare primary immunodeficiency (PID), first described in 2011[1–3] that has become gradually more recognized due to improved molecular diagnostics and increased clinical awareness.

GATA2, as a “master” transcription factor, plays a critical role in hematopoietic development[4]. Through cooperative processes that include other transcription factors, it controls the transition from hemogenic endothelium to hematopoietic stem cells and is required for survival and self-renewal of these cells[5]. GATA2 is also important for other tissue-forming stem cells, e.g., in the inner ear[6].

The heterozygous variants causing GATA2 deficiency are located both in coding, non-coding and enhancer regions[7]. The disease-causing loss-of-function variants can be localized across the gene. These variants can lead to defective DNA-binding capacity of the transcription factor and may cause disease through haploinsufficiency of the functional protein[5, 8]. Missense variants within the zink finger 2 (ZNF2) domain are the most frequent germline disease-causing *GATA2* variants [9]. It has been estimated that approximately 1/3 of the patients have an autosomal dominant inherited disease-causing variant[10], whereas the remaining have a de novo *GATA2* variant[7]. Of note, somatic variants in *GATA2* are known to be drivers of myeloid neoplasia in adults. Such variants are diverse, may cause gain-of-function effects, and be located across the whole gene. This includes missense variants in the zink finger 1 (ZNF1) domain, which has not been observed in constitutional GATA2 deficiency[8].

Typically, GATA2 deficiency becomes clinically apparent in late childhood to early adulthood. The phenotype is heterogeneous, without any clear genotype–phenotype correlation, and with an incomplete clinical penetrance[11]. Symptoms may include recurrent or severe infections, warts, cytopenia (including monocytopenia), lymphedema, alveolar proteinosis, and malignant myeloid disease[9]. Infectious complications in GATA2 deficiency are likely due to deficiency of monocytes, NK cells, and B-lymphocytes as well as defective innate immune responses, including impaired type I interferon production[12]. This leads to both increased susceptibility to viral infections (e.g., *human papilloma virus* [HPV, warts] and herpes virus infections), non-tuberculous mycobacteria, and to more common bacterial respiratory infections. Hearing loss is a common clinical feature of GATA2 deficiency and is related to the critical role of GATA2 in vestibular morphogenesis of semicircular ducts and generation of the perilymphatic space around the inner ear’s semicircular canals[6, 13]. A substantial proportion of patients develop immunodeficiency, myelodysplastic syndrome (MDS), or acute myelogenous leukemia (AML) as initial manifestation[9, 14]. GATA2 deficiency is considered the most common hereditary predisposition to pediatric MDS[15].

The only cure for GATA2 deficiency is allogeneic hematopoietic stem cell transplantation (allo-HSCT) and results are encouraging[16–20]. However, the main challenge is deciding who and when to transplant due to the complexity and inconsistency of phenotype-genotype correlation in GATA2 deficiency[9]. To further elucidate this important issue, we present detailed clinical and molecular characteristics, treatment, and outcome of 14 Norwegian patients with germline *GATA2* variants diagnosed between 2013 and 2020. The main aim of our study was to explore if detailed clinical, genetic, and bone marrow (BM) characteristics could predict end-point outcome such as death and allo-HSCT in patients with GATA2 deficiency.

## Methods

### Identification of Patients and Clinical Characteristics

The first aim of this study was to obtain a complete overview of all patients with known GATA2 deficiency in Norway. For this purpose, a network of clinical immunologists, hematologists, pediatricians, and geneticists at Oslo University Hospital (OUH) collected clinical and laboratory data on patients with GATA2 deficiency at their institution. In addition, all medical genetics departments in Norway were contacted to identify any additional GATA2 deficient individuals. Clinical information, genetic variants, treatment, and outcome were subsequently retrieved from the patients’ medical records. Patients were enrolled into the study at OUH where most of the data was obtained, while supplemental data from Patient 3 was collected at the University Hospital of North Norway, Tromsø.

### Informed Consent

Five of the patients had previously consented to be part of a genetic PID research project approved by the regional ethical committee (REC. 2014/1270–1), three patients were diagnosed with MDS in childhood and consented to be registered into the EWOG-MDS-2006 study (2015/1651/REC Nord), and all adult patients who underwent allo-HSCT had consented to publication of data (REC 11,909). In addition, due to the detailed clinical information published herein, all adult living patients signed an additional written informed consent for publication of their data and was given the opportunity to review the manuscript prior to publication. For children < 18 years, consent was given by their parents. This is in line with the recommendation given by the Ethical Constitutional board at OUH.

### Genetic Analyses

Whole exome sequencing (WES) with in silico filtering for genes causing primary immunodeficiency disorders was performed in the probands and affected relatives as part of a routine laboratory service (Patients 2, 3, 8, 9, 10, 12, and 14) or on a research basis (Patients 4, 5, 6, and 7) as previously described (Supplemental methods)[21]. Patient 1 had severe cytopenia (Table 1), and the first attempt to extract DNA from peripheral blood was not successful. A skin biopsy was therefore performed to extract DNA from fibroblasts. In parallel, peripheral blood (from puncture of the fingertip) was applied directly to a Guthrie filter card, and by using multiple filter card punches, enough DNA was extracted to run next-generation sequencing (NGS) with an amplicon-based targeted panel for constitutional variants in PID genes (Supplemental methods). By using this rapid amplicon-based method, the molecular result was available within 3 working days[22]. DNA later extracted from fibroblasts confirmed the *GATA2* variant by Sanger sequencing. Also, for Patient 13, who had advanced MDS with pancytopenia, the NGS results were available within 3 working days, with parental testing performed in parallel to evaluate as fast as possible the availability of a healthy unaffected matched related donor (MRD).

**Table 1:** Clinical characteristics and outcome in patients with GATA2 deficiency

Patient no	Family	Sex	Current age	Age at onset of symptoms/age at genetic diagnosis	Infections		Hearing loss	Hematologic abnormalities	Autoimmunity/immune dysregulation	Miscellaneous	HSCT, age	Outcome
					***Viral***	***Bacterial***						
1	A (father of P2 and P3)	M	44y	5y/41y	HPV: warts HSV: disseminated disease	Ear infections as a child	Yes	Hypoplastic BM: cytopenia, trilinear hypoplasia	No	No	41y	Alive
2	A (son of P1)	M	16y	7y/14y	HPV: warts	No	No	MDS-EB-1	No	No	16y	Alive
3	A (daughter of P1)	F	13y	8y/9y	HPV: warts	No	No	No	No	No	ND	Alive
4^a^	B (monozygotic twin to P5)	F	45y	21y/38y	HPV: warts, carcinoma in situ EBV: prolonged viremia	Recurrent respiratory inf Suppurative skin infection after BCG *	Yes	No	Progressive obliterating bronchiolitis, lupus-like syndrome	Miscarriage	39y	Alive
5^a^	B (monozygotic twin to P4)	F	† (39y)	24y/38y	HPV: warts, carcinoma VZV and EBV: prolonged viremia	Recurrent respiratory inf Suppurative skin inf. after BCG*	Yes	MDS-MLD (hypoplastic)	Progressive obliterating bronchiolitis, lupus-like syndrome	DVT × 2 Squamous cell carcinoma in the cervix, rectum, and anus	39y	Deceased 8 m post-HSCT^b^
6^a^	C	M	31y	11y/26y	No	Recurrent respiratory inf	Yes	MDS-MLD (hypoplastic)	-	Fever of unknown origin, recurrent pneumothorax	29y	Alive
7^a^	D	F	23y	6y/17y	HPV: warts	Recurrent respiratory inf	No	MDS-MLD (hypoplastic)	Interstitial lung disease	Lymphedema, acne, rosacea, rash, fatigue	22y	Alive
8	E	F	56y	0y/53y	No	No	No	Hypoplastic BM	No	Lymphedema, premature graying	ND	Alive
9	F	F	24y	15y/23y	No	No	No	AML with MDS-related changes	Erythema nodosum	DVT, PE, juvenile myoclonic epilepsy, epicanthic fold	23y	Alive
10	G (sibling to P11)	F	32y	6y/31y	HPV: warts, cervix dysplasia	Recurrent respiratory inf	Yes	MDS-MLD	No	Aneurysm of small vessels, hidradenitis suppurative, liver lesions: focal nodular hyperplasia	32y	Alive
11	G (sibling to P10)	M	† (34y)	22y/PM	No	Recurrent skin and respiratory inf	No	MDS-MLD	No	Acne, rosacea, necrotizing fasciitis, pilonidal cysts, skin infections, ulcerations	27y	Deceased 7y post-HSCT^c^
12	H	F	19y	14y/14y	No	No	Yes^d^	MDS-RCC (hypoplastic)	BPD/Asthma	Born premature (week 26 + 5), BPD	14y	Alive
13	I	M	13y	9y/11 y	HPV: warts	No	No	MDS-EB1	Asthma	Chronic skin abscesses, congenital ptosis	11y	Alive
14	J	F	31	23y/31y	No	No	Yes	MDS-SLD (hypoplastic)	No	Born prematurely (week 25), cerebral palsy, congenital hip dysplasia	Planned	Alive

Abbreviations: *AML*, acute myeloid leukemia; *BCG*, bacille Calmette Geurin; *BM*, bone marrow; *BPD*, bronchopulmonary dysplasia, *CT*, computer tomography, *HPV*, human papilloma virus; *HSCT*, hematopoietic stem cell transplantation; *Inf.*, infection; *m*, months; *MDS*, myelodysplastic syndrome; *MDS-EB1*, MDS with excess of blasts type 1; *MDS-MLD*, MDS with multilineage dysplasia; *MDS-SLD*, MDS with single lineage dysplasia; *MDS-RCC*, MDS subtype refractory cytopenia of childhood; *ND*, not done, *PM*, post mortem; *VTE*, venous thromboembolism; *y*, years, †; deceased

^a^These patients have previously been published in Stray-Pedersen, Sorte et al. 2016 (Patient 4 was 84.1, Patient 5 was 84.4, Patient 6 was 88.1, and Patient 7 was 86.1)[21]

^b^The patient was doing well after HSCT, but died unexpectedly of a cerebral hemorrhage

^c^The patient underwent lung transplantation for chronic lung GVHD 58 months after HSCT, and died of chronic lung rejection 26 months after bilateral lung transplantation

^d^The patient has reduced hearing, but this was confirmed after HSCT. Her hearing loss may be due to the disease-causing *GATA2* variant, but may also be secondary to complications of HSCT therapy, e.g., aminoglycosides

The molecular diagnosis in Patient 11 was confirmed post mortem using a BM sample collected prior to allo-HSCT (Table 2). Methods for testing for somatic occurring sequence variants on DNA extracted from whole blood or BM, and methods for testing chromosomal aberration on BM cells are described in Supplemental methods.

**Table 2:** Constitutional and acquired genetic findings in patients with GATA2 deficiency

**Patient no**	**Hematological abnormalities**	**Constitutional heterozygous variants in *GATA2*,** NM_001145661.1**,** **predicted protein effect, domain, occurrence, novelty, and reference**	**Somatic variants,** **predicted protein effect,** **VAF in BM/blood (prior to HSCT)**	**Karyotype in BM (closest to HSCT)**^**d**^	** + 8**	** − 7**
1	Hypoplastic bone marrow	c.1062_1064del, p.Thr358del, in-frame exon 6, ZNF2, likely de novo, novel variant	Unknown	46,XY[25/25]	No	No
2	MDS-EB1	c.1062_1064del, p.Thr358del, in-frame exon 6, ZNF2, paternal inherited, novel variant **Acquired germline donor variant in *GATA2* Post-HSCT**^**b**^**:** c.1215G > T, p.(Lys405Asn) missense exon 7, outside and distal to ZNF2 domain, VAF: 49,5% BM	NM_001145661.1 (**GATA2**): c.1168_1170del, p.(Lys390del), in-frame exon 7, in ZNF2, VAF: 40.2% BM NM_006758.2(**U2AF1**): c.470A>G, p.(Gln157Arg) VAF: 44,0% BM	46,XY,-7 + 8[20/20]	Yes	Yes
3	No	c.1062_1064del, p.Thr358del, in-frame exon 6, ZNF2, paternal inherited, novel variant	Unknown	Unknown	N.a	N.a
4 ^a^	No	c.1143 + 5G > A, p.Asn381fs*20, splice defect intron 6, ZNF2, de novo, novel, reported by us^c^)[21]	Unknown	Unknown	N.a	N.a
5 ^a^	MDS-MLD (hypoplastic)	c.1143 + 5G > A, p.Asn381fs*20, splice defect intron 6, ZNF2, de novo, novel, reported by us^c^[21]	None	46,XX[18/18]	No	No
6^a^	MDS-MLD (hypoplastic)	c.1078 T > A, p.Trp360Arg, missense exon 6, ZNF2, de novo, variant previously reported by others[23]	Unknown	46,XY[25/25], but FISH MYC(8q24): + 8 in 14/303 metaphases	Yes	No
7^a^	MDS-MLD (hypoplastic)	c.1061C > T, p.Thr354Met, missense exon 6, ZNF2, de novo, but a recurrent *GATA2* variant[21, 24, 25]	NM_001042749.2(**STAG2**): c.2534-2A > G, predicted splice variant with loss of acceptor site, Chr.X, VAF: 11.7% blood	47,XX, + 8[4/10]/46,XX[6/10]	Yes	No
8	Hypoplastic bone marrow	c.1017 + 1G > T, loss of donor splice site, splice defect intron 5, ZNF1, both parents deceased and not tested, novel variant	Unknown	46,XX[25/25]	No	No
9	AML with MDS-related changes	c.163C > T, p.Gln55*, nonsense exon 3, TAD domain, likely de novo (see pedigree), novel variant VAF:48.7% in BM, 49,4% in buccal swap	NM_001754.4(**RUNX1**): c.593A > G,p.(Asp198Gly) VAF:15% BM NM_156039.3(**CSF3R**): c.2326C > T, p.(Gln776*) VAF: 12.5% BM NM_032458.2(**PHF6**): c.309C > G, p.(Tyr103Ter) VAF:12.0% BM NM_033632.3(**FBXW7**): c.1513C > T, p.(Arg505Cys), VAF:11.7% BM	46,XX, der(1;7)(q10;p10), + 1[11/20]/46,XX [9/20]	No	No
10	MDS-MLD	c.1084C > T, p.Arg362*, nonsense exon 6, ZNF2, likely inherited, variant previously reported by others[15, 26–28]	NM_001123385.1(**BCOR**): c.529_530del, p.(Ser177ProfsTer8), VAF: 23.0% BM	49∼50,XX, + 6, + 8, + 21? + 21[cp7/8]/46,XX[1/8]	Yes	No
11	MDS-MLD	c.1084C > T, p.Arg362*, nonsense exon 6, ZNF2, likely inherited, variant previously reported by others[15, 26–28]	NM_015338.5(**ASXL1**): c.2324 T > G, p.(Leu775Ter) VAF: 20.5% BM NM_001042749.1**(STAG2**): c.2990 T > A, p.(Leu997Ter) VAF: 9.6% BM	47∼48,XY, + 8[10/15],der(16)t(1;16)(q21;q24[10/15], + der(16)t(1;16)[1/15], + 21[6][cp11/15]/46,XY[3/15] Trisomy 8, evolving to unbalanced 1;16 translocation and later Trisomy 21	Yes	No
12	MDS-RCC	c.1098_1100delGGA, p.Asp367del, in-frame exon 6, ZNF2, de novo, novel variant	None	46,XX,-7, + 8[15/20]	Yes	Yes
13	MDS-EB1	c.1021_1024insGCCG, p.Ala342Glyfs*43, frameshift exon 6, ZNF1 de novo, variant previously reported[29]	NM_015338.5(**ASXL1**): c.1854dupA, p.(Ala619SerfsTer16), VAF:17.0%, BM NM_015559.2 (**SETBP1**): c.2612 T > C, p.(Ile871Thr), VAF: 16.3%, BM	45,XY,-7[12/12]	No	Yes
14	MDS-SLD (hypoplastic)	c.1114G > A, p.(Ala372Thr), missense exon 6, ZNF2, variant previously reported [14]	NM_001042749.1(**STAG2**): c.707del; p.(Asn236IlefsTer20) VAF: 5.1%, BM	46,XX[25/25]	No	No

Abbreviations: *AML*, acute myeloid leukemia; *BM*, bone marrow; *Chr*, chromosome; *HSCT*, hematopoietic stem cell transplantation; *MDS*, myelodysplastic syndrome; *MDS-EB1*, MDS with excess blasts type 1; *MDS-MLD*, MDS with multilineage dysplasia; *MDS-RCC*, MDS subtype refractory cytopenia of childhood; *MDS-SLD*, MDS with single lineage dysplasia; *N.a.*, Not applicable; *TAD*, N-terminal transactivation domain, *ZNF2*, Zinc finger 2 domain in GATA2 protein; *VAF*, variant allele frequency

^a^These patients have previously been published in Stray-Pedersen, Sorte et al. (2016) (Patient 4 was 84.1, Patient 5 was 84.4, Patient 6 was 88.1, and Patient 7 was 86.1)[21]

^b^This disease-related GATA2 variant was detected in a routine BM at day + 28 post-HSCT; it turned out to be donor-derived (from a MUD)

^c^WES identified a potential splicing variant in *GATA2* (c.1143 + 5G > A) in Patient 4. The variant was predicted (Alamut®) to inactivate the donor site of *GATA2* exon 5. PCR amplification of *GATA2* exon 4 to 7 on cDNA showed that most transcripts were normally spliced resulting in a main product of ~ 400 bps, as seen in the normal control. A slightly longer PCR product including 64 bps of intron 6 sequence via a cryptic donor site in intron 6 (NM_001145661.1), was observed in the sample from Patient 4, but not in the control (see Supplementary information). Sanger sequencing identified the *GATA2* splicing variant in the proband’s deceased monozygotic twin (Patient 5). Details described in Supplemental Figure E6 in Stray-Pedersen, Sorte et al. (2016)[21]

^d^Nomenclature according to ISCN (The International System for Human Cytogenetic Nomenclature) 2020 guidelines

## Results

### Characteristics of Patients

Between 2013 and 2020, ten index cases, or probands, and four additional symptomatic patients with germline *GATA2* variants were identified (9 females, 5 male, Table 1). Five adult patients were diagnosed by infectious disease specialists (Patients 1, 4, 5, 10, and 11) where infections (mostly HPV infection/warts and recurrent bacterial airway infections) were prominent symptoms. Four additional adult patients were identified by hematologists (Patients 6, 8, 9, and 14), where three were referred with pancytopenia and one patient had AML (also with pancytopenia). Three patients were diagnosed by pediatricians, two patients with MDS (Patients 12 and 13) and one patient with extensive warts and NK-/B-cell deficiency (Patient 7). Additionally, two patients with GATA2 deficiency were identified after family screening (Patients 2 and 3). We did not detect any asymptomatic individuals with GATA2 deficiency in this study.

The mean age for debut of symptoms, that we considered related to GATA2 deficiency, was 12 years (range 0–24 years, Supplemental Table S1). The median time from these symptoms to a diagnosis of GATA2 deficiency was 11 years, range 0–53 years (Supplemental Table S1). Retrospectively, hearing loss, warts, and skin manifestations were the most common early symptoms, which in some patients became apparent many years before the genetic diagnosis of GATA2 deficiency was made (Supplemental Table S1).

A summary of the patients’ clinical characteristics is given in Table 1. Viral infections such as HPV-associated warts were common, affecting eight patients. In addition, two patients had disseminated BCG infections (after vaccination), and one patient had a life-threatening disseminated HSV infection (originating from genitalia and disseminating to CNS and liver). Two patients experienced prolonged EBV and/or *Varicella zoster* viremia. Furthermore, six patients had recurrent bacterial airway infections. Interestingly, one patient had early graying (Patient 8), with normal telomere length, and one patient had multiple aneurysms of small vessels (coronary arteries, axillary arteries, and an iliac artery; Patient 10), which both represent clinical characteristics not previously described in GATA2 deficiency. In Patient 10, *Varicella zoster* infection was excluded as a cause of vasculitis with negative VZV PCR in blood. In addition, two patients had obliterating bronchiolitis (Patients 4 and 5), which has been reported in only one previous patient with GATA2 deficiency [30].

Affected cell lineages and immunoglobulin levels prior to allo-HSCT are listed in Table 3. As expected, the majority of patients had decreased levels of monocytes (11/14) and one patient had increased levels of monocytes (Patient 12). In addition, decreased levels of B cells (10/11) and NK cells (9/11) were common findings (three patients did not have NK- and B cells measured before allo-HSCT).

**Table 3:** Immunoglobulin levels and affected cell lineage in peripheral blood prior to HSCT

Patient no	Affected cell lineage (normal range)							Immunoglobulins		Time before HSCT (months)
	*CD19* + *,* *cells* × *10*^*6*^*/L* *(100–500)*	*NK* *cells* × *10*^*6*^* /L* *(100–400)*	*CD3* + *T- cells* × *10*^*6*^*/L* *(800–2400)*	*CD4* + *T- cells* × *10*^*6*^*/L* *(500–1400)*	*CD8* + *T-cells* × *10*^*6*^*/L* *(200–2000)*	*Monocytes* × *10*^*9*^*/L* *(0.2–0.8)*	*Neutrophils* × *10*^*9*^*/L* *(1.5–7.3)*	*IgG* *g/L* *(6.9–15.7)*	*IgG2* *g/L* *(1.69–15.7)*	
1	**0**	**0**	**118**	**43**	**64**	**0.0**	**0.2**	**5.9**	ND	5
2	**10**	**10**	**550**	**200**	300	0.3	2.9	**9.0**	**ND**	4.5
3	160	150	1930	780	950	0.2	2.4	8.4	ND	NA
4	** < 10**	**15**	**349**	**185**	**145**	**0.0**	3.4	9.5	**1,24**	17
5	**6**	**1**	**259**	**134**	**66**	**0.0**	6.0	13.0	**0.79**	22^1^
6	**2**	**0**	809	**495**	326	**0.1**	**0.5**	**44.3**^2^	2.3	4
7	**18**	**19**	913	545	341	**0.0**	2.8	14.0	2.60	3
8^3^	**40**	**2**	1335	546	761	**0.1**	1.9	9.7	ND	NA
9	ND	ND	ND	ND	ND	**0.0**	4.2	**16.2**	-	1
10	**70**	206	**247**	**134**	**108**	**0.1**	1.7	**18.5**	**0.79**	6
11	ND	ND	ND	ND	ND	**0.0**	**0.9**	9.5	2.11	10
12	ND	ND	ND	ND	ND	**1.7**	2.6	8.1	ND	1
13^4^	**28**	**13**	1073	633	417	**0.2**	0.3	12.8	ND	0.5
14	**8**	**11**	**676**	**323**	341	**0.1**	**1.2**	11.9	**1.14**	NA

Abnormal values are given in bold

^1^On Prednisolone 20 mg a day when these samples were taken

^2^Hypergammaglobulinemia on IVIG due to IgG2 deficiency

^3^The values are from the time at diagnosis of GATA2 deficiency 3 years ago

^4^The reference values for Patient 13 who was 12 years old at the time of HSCT are CD19 200–600, NK 70–1200, CD3 800–3500, cd4 400–1200, cd8 200–1200 (given in cells × 10^6^/L) and for IgG the normal reference value was 6.1–14.9 g/L

*ND*, not done; *NA*, not applicable

### Germline GATA2 Variants and Somatic Variants in Other Genes

Ten different *GATA2* pathogenic, or likely pathogenic, variants were found in 14 patients (Table 2). All identified constitutional *GATA2* variants, except one, were located in the ZNF2 domain, corresponding to or in close proximity to exons 5 and 6 (Table 2). Three nonsense variants (p.Ala342Glyfs*43, p.Arg362*, p.Asn381fs*20), one + 1 splicing variant, three missense variants (p.Thr354Met, p.Trp360Arg, and p.Ala372Thr), all previously reported to be disease-causing[14, 23–25], and two novel in-frame deletions (p.Thr358del, p.Asp367del) were found. The variant located in exon 3, outside the ZNF2 domain, was a nonsense variant (c.163C > T, p.Gln55*). It was initially identified by the NGS myeloid panel with variant allele frequency (VAF) 49% in the DNA from the patient’s BM and later verified to be germline with VAF 49% in a buccal DNA sample (Patient 9, Table 2).

Patient 2 had a paternal inherited in-frame deletion, c.1062_1064del (p.Thr358del), in the ZNF2 domain, and a somatic in-frame deletion, c.1168_1170del (p.Lys390del), with a fairly high VAF, 40.2% in BM. As expected, these two in-frame deletions were no longer detectable after allo-HSCT. Surprisingly, in the first post-transplant BM sample at day + 28, we detected another acquired *GATA2* variant, c.1215G > T (p.Lys405Asn) with VAF 49.5%. This missense mutation variant, affecting an amino acid located C-terminal to the ZNF2 domain, is a variant of unknown clinical significance. It is most likely a rare benign variant, which in retrospect was confirmed to be constitutional in the unrelated BM donor (Table 2). It is evaluated to ACMG category 3 minus, since altogether 5 heterozygote individuals with the same amino acid change p.Lys405Asn are reported in GnomAD (v.2.1.1)[31]. As far as we know, missense variants located outside the ZNF2 domain rarely represent constitutional susceptibility to development of myelodysplasia. One exception is the p.Ser447Arg located C-terminal of the ZNF2 domain[32], while no other missense variants outside the ZNF2 domain are currently defined as pathogenic or likely pathogenic in ClinVar **(**www.clinvar.com**)** as of year 2021. Karyotype abnormalities and somatic variants in other genes observed in the 14 patients are presented in Table 2. Trisomy 8 (*n* = 6), monosomy 7 (*n* = 3), *STAG2* variants (*n* = 3), *ASXL1* variants (*n* = 2), a combination of somatic variants in *RUNX1/CSF3R/PHF6/FBXW7* (*n* = 1), and variants in the following MDS genes[33] were observed once in separate individuals: *BCOR*, *SETBP1*, *U2AF1*, and somatic *GATA2*. One adult GATA2 deficient patient who developed AML had an unbalanced translocation der(1;7) in the leukemic clone.

Since GATA2 deficiency is considered the most common hereditary predisposition to pediatric MDS, we estimated the proportion of pediatric patients diagnosed with MDS in Norway that had a germline *GATA2* variant in the same time period (2013–2020). We found that three out of 14 pediatric patients diagnosed with MDS (21%) had GATA2 deficiency. Of note, these are 14 pediatric MDS patients and not the same cohort of 14 GATA2 deficient patients described above (except three overlapping pediatric patients, Patients 2, 12, and 13, with MDS). Two of the 3 pediatric GATA2 deficient patients with MDS had both monosomy 7 and trisomy 8 in their bone marrow cells.

Patients 4, 5, 8, 9, 10, and 11 from Family B, E, F, and G had frameshift or other definitive loss-of-function variants, while Patients 1, 2, 3, 6, 7, 12, 13, and 14 from Family A, C, D, H, I, and J had in-frame deletions or missense variants. No specific genotype–phenotype correlations were found in our data set, i.e., regarding debut of symptoms, type and distribution of infections, age of transition to MDS/AML, somatic occurring variants in blood and BM. The severity of the clinical presentations also varied within families.

### Families and Predictive Genetic Testing

**Fig. 1:**
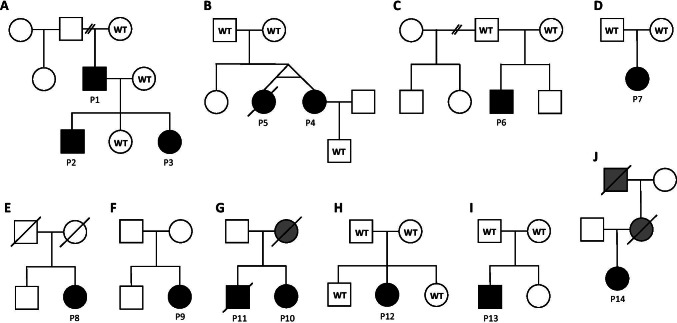
Pedigrees of the ten families, including 14 patients, with known GATA2 deficiency. Solid symbols denote affected status. Individuals marked in gray are deceased and not tested for GATA2 deficiency but are suspected to carry the disease-causing variant. In family G, the mother of Patients 10 and 11 died at age 30 of acute respiratory distress syndrome, 27 years ago. She also had lymphedema since birth. In light of their mother’s medical history, the GATA2 variant is probably maternally inherited. The father is alive and healthy. In family J, the mother of Patient 14 had a combined B and T cell defect, warts, myelodysplastic syndrome, lymphedema, and recurrent respiratory tract infections. She died of vulval cancer at the age of 38. The maternal grandfather of Patient 14 died of acute leukemia at the age of 33. WT, wild-type

The pedigrees of the 10 families are presented in detail in Fig. 1. Patient 1 (Family A) had three apparently healthy children, when he was diagnosed with GATA2 deficiency. After genetic testing of first-degree relatives, we found that two of his children (Patients 2 and 3) had inherited the *GATA2* variant. For Patient 2, initial clinical work-up revealed only mild cytopenia and warts. However, within 2 years of follow-up, he developed pancytopenia and transfusion dependency and was diagnosed with MDS-EB1. His sister, Patient 3, has warts as her only clinical manifestation, but will be followed up regularly for development of cytopenia/MDS.

In Family G, two siblings had the same germline *GATA2* variant (Patients 10 and 11). Their mother died 27 years ago, at the age of 30, of acute respiratory distress syndrome (ARDS), of unknown etiology. She also had lymphedema since birth. In light of their mother’s medical history with lymphedema and ARDS, which could be secondary to complications related GATA2 deficiency, the *GATA2* variant is probably maternally inherited. Their father is alive and healthy.

The deceased mother of Patient 14 (Family J) had a combined B- and T cell defect, warts, MDS, lymphedema, and recurrent respiratory tract infections. At the age of 38 (years), she died of metastatic vulval cancer. The maternal grandfather of Patient 14 died of acute leukemia at the age of 33. Considering the family history, it is very likely that Patient 14 had inherited her germline *GATA2* variant from her maternal grandfather via her mother. Both individuals died before GATA2 deficiency was acknowledged as a cause of PID. Her mother’s siblings are now offered genetic counselling/testing for GATA2 deficiency.

### Allo-HSCT in Patients with GATA2 Deficiency

Twelve of 14 (86%) patients with GATA2 deficiency were found to have a clinical indication, cytogenetic findings, and/or molecular findings warranting to proceed to allo-HSCT. As of today, 11 patients have undergone allo-HSCT, whereas one is recently accepted for allo-HSCT (Patient 14). Clinical features that lead to the decision to perform allo-HSCT were previous life-threatening disseminated *HSV* infection (Patient 1), severe obliterating bronchiolitis and in situ carcinoma (Patients 4 and 5), MDS with cytogenetic abnormalities (monosomy 7) and/or excess of blasts with high likelihood of progression to leukemic transformation (Patients 2, 6, 7, 9, 12, 13, and 14), MDS and warts with high-grade dysplasia (Patient 10), and symptoms of severe immunodeficiency and MDS (Patient 11). Details on the allo-HSCT procedure, including conditioning, donor selection, stem cell source, donor/recipient cytomegalovirus status, donor chimerism, graft versus host disease (GVHD) prophylaxis, and the occurrence of GVHD, are presented in Table 4.

**Table 4:** HSCT details for eleven patients with GATA2 deficiency

Patient no	Age at HSCT	Donor	Stem cell source	HLA Match	CD34 + , × 10^6^/kg	CMV status d/r	Conditioning regimen & In vivo T-cell depletion	Chimerism %*Day* + *28*	GVHD prophylaxis	GVHD	Complications
1	41 y	MUD	PBSC	10/10 (11/12)	7.8	-/ +	RIC: Fludarabine 150 mg/ m^2^, Busulfan 8 mg/kg, ATG Thymoglobulin 4 mg/kg	99%	Mtx + CsA	No	Hemorrhagic cystitis (BK-virus)
2	16 y	MUD	PBSC	10/10 (10/12)	9.7	-/ -	MAC*: Busulfan for 4 days (TDM; Css 825 ng/ml), Cyclophosphamide 120 mg/kg, Melphalan 140 mg/m^2^, ATG Grafalon 3 × 10 mg/kg	> 99%	Mtx + CsA	No	*E. coli* sepsis; BK-virus cystitis; mucositis (grade 3)
4	39 y	MUD	PBSC	10/10 (12/12)	10.6	+ / +	RIC: Fludarabine 150 mg/ m^2^, Busulfan 8 mg/kg. ATG Thymoglobulin 4 mg/kg	> 99%	Mtx + CsA	Chronic: skin and gut	CMV reactivation
5	39 y	MUD	PBSC	10/10 (11/12)	5.2	+/ -	RIC: Fludarabine 90 mg/m^2^, 2 Gy TBI	99%	Mtx + CsA	No	*Enterococcus faecalis* sepsis (2 months post-HSCT), prolonged cytopenia, died of intracerebral hemorrhage 8 months post-HSCT
6	29 y	MRD	PBSC	HLA-id sibling	5.4	+ / +	RIC: Fludarabine 150 mg/m^2^, Busulfan 8 mg/kg	98%	Mtx + CsA	Chronic: liver, oral mucosa and genitalia	Cytopenia at day + 33, osteoporosis, compression fractures
7	22 y	MUD	PBSC	10/10	6.9	-/ -	MAC: Fludarabine 150 mg/m^2^, Treosulfan 42 g/m^2^, ATG Thymoglobulin 4 mg/kg	> 99%	Mtx + CsA	Chronic (limited): skin	*Moraxella nonliquefaciens* sepsis on day 0. *E. coli* sepsis day + 14. Oral mucositis grade IV. PTLD 7 weeks post-HSCT
9	23 y	MUD	PBSC	10/10 (11/12)	4.3	+/ -	MAC: Fludarabine 160 mg/m^2^, Busulfan 12,8 mg/kg (i.v.), ATG Thymoglobulin 4 mg/kg	> 99%	Mtx + CsA	Acute GvHD grade I: skin	None
10	32 y	MUD	PBSC	10/10 (11/12)	5.9	-/ -	MAC: Fludarabine 150 mg/m^2^, Treosulfan 42 g/m^2^, ATG Thymoglobulin 4 mg/kg	> 99%	Mtx + CsA	Acute GvHD: serositis	None
11	27 y	MUD	PBSC	10/10 (10/12)	10.2	-/ -	MAC: Cyclophosphamide 100 mg/kg, Busulfan 16 mg/kg	N.a	Mtx + CsA	Chronic (extensive): gut, eye, and lung	Hemorrhagic cystitis, Herpes oesophagitis
12	14 y	MUD	BM	10/10 (11/12)	TNC: 3.5 × 10^8^/kg	-/ -	MAC*: Fludarabine 160 mg/m^2^, Treosulfan 42 g/m^2^,Thiotepa 8 mg/kg, ATG Grafalon 3 × 10 mg/kg	day + 84: > 99%	Mtx + CsA	No	Impetigo day + 40
13	11 y	MMFD (father)	PBSC, TCRab + /CD19 + depletion in vitro	Haploidentical	10.3	+ / +	MAC*: Fludarabine 160 mg/m2, Thiotepa 10 mg/kg, Melphalan 140 mg/m^2^, ATG Grafalon 3 × 10 mg/kg	> 99%	MMF (until day + 28)	No	None

Abbreviations: *BM*, bone marrow; *CMV*, cytomegalovirus; *CsA*, cyclosporine A; *Css*, concentration at steady-state; *Cya*, cyclosporine A; *GVHD*, graft versus host disease; *i.v.*, intravenous; *MAC*, myeloablative conditioning; *MMF*, mycophenolate mofetil; *MMFD*, mismatched family donor; *MRD*, matched related donor; *Mtx*, methotrexate: *MUD*, matched unrelated donor; *N.a.*, not available; *PBSC*, peripheral blood stem cells; *RIC*, reduced-intensity conditioning; *TDM*, therapeutic drug monitoring; *Tx*, transplantation

^*^According to HSCT recommendations by the EWOG-MDS study group

### Clinical Outcome

The clinical outcome of all 14 patients is presented in Table 1.

Two adult patients (18%) died after allo-HSCT. Patient 5 had persistent thrombocytopenia and died of a cerebral hemorrhage 8 months post transplantation. Patient 11 developed respiratory failure due to cGVHD in the lungs and received a bilateral pulmonary transplant 5 years post allo-HSCT. However, he developed chronic pulmonary rejection and died 2 years after lung transplantation and 7 years after allo-HSCT. The mean follow-up of the nine patients still alive after allo-HSCT is 26 months (range 3–78 months). The incidence of aGvHD and cGVHD among the eleven transplanted patients was 25% and 33%, respectively, all occurring in patients > 18 years of age (Table 4). None of the pediatric patients had experienced aGVHD or cGVHD, serious infectious complications, or any serious or unexpected transplant-related acute or late toxicity. Their transplantation courses were uneventful and did not principally differ from MDS patients without germline disease-causing *GATA2* variants.

One patient is listed for allo-HSCT (Patient 14) and two patients are followed regularly in the out-patient clinic (Patients 3 and 8).

## Discussion

This retrospective study describes clinical features and outcome of 14 patients from ten families diagnosed with GATA2 deficiency in Norway. The main findings were as follows: (i) We found a diverse clinical phenotype dominated by cytopenia (13/14), myeloid neoplasia (MDS/AML) (10/14), warts (8/14), and hearing loss (7/14). (ii) We observed two novel clinical features multiple aneurysms of small vessels (*n* = 1) and early graying (*n* = 1) that could be associated with GATA2 deficiency. (iii) The majority of patients (11/14) had already undergone allo-HSCT at the time of our analysis, illustrating the need for allo-HSCT in a large proportion of patients with GATA2 deficiency in Norway, and most likely in other countries. (iv) Genetic testing should be offered to first-degree relatives, particularly children, to identify individuals with GATA2 deficiency that need close surveillance.

Genetic testing performed as soon as a clinical suspicion is raised, increases the likelihood of an early and correct molecular diagnosis. In Norway, exome-based panels including *GATA2* are offered as a routine diagnostic laboratory service for constitutional hematological disorders and PID [21]. However, this approach will not detect all *GATA2* variants. Germline variants located in the intronic transcriptional enhancer elements, the *cis*-acting E-box/GATA and ETS motifs within intron 5 (NM_001145661.1), may cause GATA2 deficiency[34]. Supplementary Sanger sequencing of the enhancer element sequences in intron 5[34] was only performed in one of the laboratories (see Supplementary Methods). Despite supplementary copy number variant calling from exome data, with chromosomal microarray and MLPA only in selected patients (Supplementary Methods), some structural variants may go undetected. In addition, synonymous disease-causing *GATA2* variants resulting in selective loss of mutated RNA were recently reported [35]. Disease-causing intronic variants, small intragenic variants, such as structural variants and synonymous variants, may also have escaped detection and hence, individuals with GATA2 deficiency may have been overlooked.

With the recent introduction of targeted NGS panels in the work-up of myeloid neoplasms searching for somatic *GATA2* variants, unexpected germline variants can be identified, which may reveal the underlying constitutional cause of the myeloid disease and increase the prevalence of known GATA2 deficiency. Improving the NGS panels targeting both germline and somatic variants by deeper coverage of the whole genes including important non-exonic regions, better algorithms for detection of structural variants, and attention to rare synonymous variants may enhance the identification of GATA2 deficiency.

In this case series, we found two clinical features that have not yet been described in GATA2 deficiency, namely small vessel aneurysm and early graying. Multiple small vessel aneurysms found in Patient 10 may be secondary to vasculitis, or could also represent a novel vascular feature associated with GATA2 deficiency. In fact, it has been suggested that alteration in GATA2 expression may be of importance for vascular integrity[36]. The observation of premature graying may be a coincidence. In the absence of telomere biology disorders as in the present patients, one may speculate if this feature may reflect a GATA2-linked autoimmune phenomenon which has gone undetected.

One of the aims of this retrospective study was to describe the clinical characteristics, *GATA2* variants, and other molecular variants representing risk factors for clonal evolution that could aid us in the difficult decision regarding: “Who and when to transplant”? The high proportion of patients (79%) that had already undergone allo-HSCT in our cohort was somewhat surprising. Donadieu et al. have published the largest cohort of patients with GATA2 deficiency, and found that only 28 patients (35%) of 79 patients had undergone allo-HSCT. However, this low percentage of allo-HSCT did not correspond with the severity of the disease in this cohort. At the age of 40, the authors reported a mortality rate of 35% and a hematological malignancy rate of 80%[14]. The high proportion of allo-HSCT in our study may have been influenced by increased awareness of negative prospective clinical markers of GATA2 deficiency. Based on the findings from our study and the high morbidity and mortality rate reported by Donadieu et al., it is clear that these patients need to be monitored closely. Ideally, allo-HSCT should be performed before they develop malignancies (both solid tumors and hematological malignancies)[37] or severe/recurrent infections causing organ failure. In our opinion, a history of disseminated viral infection, aggressive HPV infection (particular with dysplasia), or myeloid clonal disease is clear indication to consider allo-HSCT[14]. First-degree relatives with a severe outcome of the disease may further strengthen the indication for an early allo-HSCT in symptomatic patients with GATA2 deficiency. Overall, the decision to perform an allo-HSCT requires careful weighing of potential gain (restore immune function; diminish the risk of hematological malignancies) versus possible transplant complications, including GVHD and transplant-related mortality. This is particularly challenging given the lack of genotype–phenotype correlation. Keeping in mind that the observation time is short for some of the patients in our study, the survival rate after allo-HSCT was 82% (9/11). In patients with GATA2 deficiency, previous publications have reported 86% survival 2 years after HSCT (*n* = 22)[16], 73% and 62% survival 1 and 5 years after HSCT, respectively (*n* = 28)[14], 72%, 65%, and 54% survival 1, 2, and 4 years after HSCT, respectively (*n* = 21)[9], and 57% 3, 5 years after HSCT (*n* = 14)[38]. These cohorts are, however, not necessarily comparable in terms of severity of disease and conditioning regimen.

Two children were diagnosed with GATA2 deficiency after family screening. The hematological surveillance of one of these children led to detection of hematological abnormalities consistent with MDS and, in the end, a timely allo-HSCT. We therefore recommend genetic testing of children of affected adults and hematological surveillance of individuals with known pathogenic germline *GATA2* variants. This includes annual BM investigations with morphological and cytogenetic evaluations, and testing with NGS myeloid panel to screen for somatic occurring molecular drivers of malignancies. Monosomy 7 and trisomy 8 have been reported by others to be the major cytogenetic aberrations in hematopoietic cells of patients with GATA2 deficiency and MDS[15]. Advanced MDS disease and monosomy 7 have been related to worse outcome, especially for pediatric patients with *GATA2* germline disease[20]. We found monosomy 7 only in ¼, but trisomy 8 in half of the karyotyped patients, which is in line with a previous published study by McReynolds et al. [39]. Two patients in our GATA2 cohort had monosomy 7 and trisomy 8, both were children. Some of the other somatic variants in our cohort occurred in genes previously reported to be mutated in GATA2 deficiency with MDS, such as *ASXL1*[40], *STAG2*, *SEPTBP1*, and *RUNX1*[41, 42]. Interestingly, one adult patient with GATA2 deficiency and MDS-related AML had a der(1;7) in the leukemic clone, a translocation that has recently been shown to be enriched in pediatric MDS patients with germline GATA2 mutations[43]. However, our number of patients are too small to determine possible genotype–phenotype correlations related to clonal disease progression.

Previous reports have suggested that plasma levels of FLT3LG can be used as predictor of hematological disease in GATA2 deficiency, and used in clinical monitoring post-HSCT [25]. Unfortunately, we lack serum or plasma samples taken before and after HSCT in our cohort, and the role of FLT3LG as a disease progression marker could be explored in future studies. Our main conclusion of this study is that the majority of patients with symptomatic GATA2 deficiency will need allo-HSCT, and close surveillance of these patients is important to find the “optimal window” for allo-HSCT. We advocate a more offensive approach to allo-HSCT than previously described.

## Supplementary Information

Below is the link to the electronic supplementary material.

Supplementary file1 (DOCX 55 KB)

## Data Availability

Upon request.

## Abbreviations

aGVHD: Acute graft versus host disease
allo-HSCT: Allogeneic hematopoietic stem cell transplantation
AML: Acute myelogenous leukemia
ARDS: Acute respiratory distress syndrome
BM: Bone marrow
cGVHD: Chronic graft versus host disease
GVHD: Graft versus host disease
HPV: Human papilloma virus
MDS: Myelodysplastic syndrome
MRD: Matched related donor
MUD: Matched unrelated donors
NGS: Next-generation sequencing
PID: Primary immunodeficiency
VAF: Variant allele frequency
WES: Whole exome sequencing
ZNF1: Zink finger 1
ZNF2: Zink finger 2
