# Supplementary material for: A Nationwide Study of GATA2 Deficiency in Norway—the Majority of Patients Have Undergone Allo-HSCT
Source: J Clin Immunol. 2021 Dec 10;42(2):404–20. doi: 10.1007/s10875-021-01189-y (PMC8664000; doi:10.1007/s10875-021-01189-y)
Supplement: Supplementary file 1 — Supplementary file1 (DOCX 55 KB) [file 10875_2021_1189_MOESM1_ESM.docx]

**Supplemental data**

**A nationwide study of GATA2 deficiency in Norway – the majority of patients have undergone allo-HSCT**

**Silje F. Jørgensen, Jochen Buechner, Anders E. Myhre, Eivind Galteland, Signe Spetalen, Mari Ann Kulseth, Hanne S. Sorte, Øystein L. Holla, Emma Lundman, Charlotte Alme, Ingvild Heier, Trond Flægstad,** Yngvar Fløisand, **Andreas Benneche , Børre Fevang , Pål Aukrust, ^#^Asbjørg Stray-Pedersen, ^#^Tobias Gedde-Dahl, ^#^Ingvild Nordøy**

**^#^Contributed equally**

**Supplemental methods**

**Supplemental Table**

**Supplemental methods:**

*Gene panels based on exome sequencing*

Whole exome sequencing (WES) with *in silico* filtering for genes causing primary immunodeficiency disorders was performed in the probands and affected relatives as part of a routine laboratory service as previously described(1). The typical read-depth obtained with WES was 100x. The gene panels used for filtering were version 03 “NGS-Immunodeficiency” at the Department of Medical Genetics*,*Oslo University Hospital (OUH) and version 07 “NGS-primary immunodeficiency and -dysfunction” at SH-TEL (the Department of Medical Genetics*,* Telemark Hospital, Skien); updated gene lists are available online (<https://genetikkportalen.no/>). Internally developed informatics pipelines at OUH and SH-TEL were used for analysis of these next generation sequencing (NGS) data. Copy number variant (CNV) calling at OUH was performed from the WES data as previously described (1).

Chromosomal microarray was performed in primary immunodeficient patients without findings on the WES based panel, or when CNV prediction data indicated the presence of a relevant CNV. The chromosomal microarray was performed with a custom 1M Agilent high resolution exonic focused oligoarray (1). MLPA (Multiplex Ligation-dependent Probe Amplification) using SALSA^®^MLPA^®^Probemix P437-B1 was performed at SH-TEL when *GATA2* deficiency was suspected. Starting in 2019, these patients were also subject to Sanger sequencing of Chr3(GRCh38):128483445-128482873 containing the intron 5 enhancer element, but only at SH-TEL.

*Testing for somatic variants with amplicon-based sequencing and karyotyping*

Testing for somatic occurring sequence variants was performed on DNA extracted from whole blood or BM by The Illumina’s TruSight Myeloid Sequencing Panel (Illumina, San Diego, CA, USA). The Illumina’s TruSight Myeloid Sequencing Panel is a PCR-amplicon based targeted NGS-panel which according to the company’s information, covers 15 full genes (exons only) and 39 additional genes that are oncogenic hotspots. Alignment and variant calling were performed against GRCh37/hg19 with MiSeq Reporter Software v.2.6.2.3 (Illumina). SNV’s and indels <60 bp were annotated by VariantStudio v.3.0*.* The median number of reads was 6500-8700 with myeloid panel. Testing for chromosomal aberrations was performed on BM cells cultured by standard methods for cytogenetic evaluation under the microscope. Chromosome preparations were made from metaphase cells of a 24-hours culture, G-banded using Leishman stain, and karyotyped according to The International System for Human Cytogenetic Nomenclature ISCN 2020 guidelines. Chromosomal microarray was not performed as part of the testing for somatic occurring chromosomal aberrations.

*Rapid amplicon-based sequencing after DNA extraction from dry blood spots*

NGS with amplicon-based targeted panel for constitutional variants was performed in Patient 1 and 13 on blood samples collected on Guthrie filter cards and analysed in the Newborn screening laboratory, as part of an ongoing research project (REC. 2014/1270-1). Samples were punched from the filter card using a Panthera-Puncher 9 (Perkin Elmer, Turku, Finland). DNA was extracted from a 3.2 mm punch of one dry blood spot on the filter card, and one punch contains on average 3μL blood. The manual method for DNA extraction from filter card blood is published in detail elsewhere(2). DNA was analysed using the Ion AmpliSeq library kit with the Thermo Fisher predesigned gene panel PIDv2 containing 264 primary immunodeficiency disease genes, and then sequenced on a benchtop ION-PGM (Thermo Fisher Scientific)(3). The average number of reads were 400x with Ampliseq NGS. The annotated variant calling file was filtered in Ion Reporter^TM^ Software, which was also used for presentation and evaluation of the NGS data. Total turn-around-time from sampling to final result can come down to 2-3 working days(3). Of note, neither the gene panels based on exome sequencing, the TruSight Myeloid panel or the predesigned PIDv2 Ampliseq NGS panel included the intron 5 region in *GATA2*, Chr3(GRCh38):g.128483330-128483288, where the intronic enhancer elements are located(4).

*Gene variant evaluation*

The BAM files were visualized in Integrative Genomics Viewer(5) and Alamut Visual (v.2.11, Interactive Bioinformatics, France).Variant evaluation and classification were performed according to the ACMG guidelines(6). The assumed pathogenic variants identified by NGS were confirmed using Sanger sequencing, and segregation testing of the parents and affected family members were performed with Sanger sequencing.

*mRNA analysis of c.1143+5G<A*

A blood sample from the index patient was collected into PAXgene Blood RNA tubes and total RNA was extracted using the PAXgene Blood RNA kit (Qiagen, Valencia, CA, USA). RT-PCR was performed using Qiagen OneStep RT-PCR kit (Qiagen, Valencia, CA, USA) and primers located in exon 4 (5’-GCAAGGCTCGTTCCTGTTCA-3’) and exon 7 (5’-CACTTTGACAGCTCCTCGAA-3’). The amplified products were analyzed on an Agilent 2000 BioAnalyzer (Agilent Technologies, Palo Alto, CA) and sequenced using the BigDye Terminator v3.1 Cycle Sequencing Kit (Applied Biosystems, Foster City, CA, USA). Data were evaluated by using the sequencing analyses software Sequencing Analyses 5.2 (Applied Biosystems, Foster City, California, USA).

The RT_PCR revealed two products


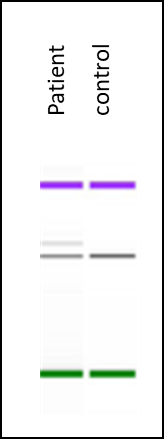


The sequencing of the upper band in the patient sample showed an insertion of 64bp from intron 6 (shown in small letters)

TCGGCCGCCAGAAGAGCCGGCACCTGTTGTGCAAATTGTCAGACGACAACCACCACCTTATGGCGCCGAAACGCCAACGGGGACCCTGTCTGCAACGCCTGTGGCCTCTACTACAAGCTGCACAATgtgagtgcgccccgccccggccaccccgcccctcccaggggacctctgcgctttgtgctgccagGTTAACAGGCCACTGACCATGAAGAAGGAAGGGATCCAGACTCGGAACCGGAAGATGTCCAA

This insertion causes a loss of reading frame and insertion of a premature termination codon, p.Asn381fs*20

**Supplemental Table S1**

| **Patient ID** | **Initial symptoms, age (years)** | **Age at diagnosis, years** | **Time from onset of symptoms to diagnosis, years** |
| --- | --- | --- | --- |
| 1 | Hearing loss and warts, 5 | 41 | 36 |
| 2 | Warts, 7 | 14 | 7 |
| 3 | Warts, 8 | 9 | 1 |
| 4 | Exanthema, 21 | 38 | 17 |
| 5 | Erythema nodosum, 24 | 38 | 14 |
| 6 | Cytopenia, 11 | 26 | 15 |
| 7 | Warts, 6 | 17 | 11 |
| 8 | Innate lymphedema, 0 | 53 | 53 |
| 9 | Epilepsy, 15 | 23 | 8 |
| 10 | Deafness, 6 | 31 | 25 |
| 11 | Acne, 22 | PM^a^ | - |
| 12 | MDS and cytopenia^b^, 14 | 14 | 0 |
| 13 | Acne, 9 | 11 | 2 |
| 14 | Hearing loss, 23 | 31 | 8 |

PM: Postmortem, ^a^ Diagnosis made on BM 1,5 year after his death. He died at age 34. ^b^ She also experienced fever after HPV vaccine

**References**

1. Stray-Pedersen A, Sorte HS, Samarakoon P, Gambin T, Chinn IK, Coban Akdemir ZH, et al. Primary immunodeficiency diseases: Genomic approaches delineate heterogeneous Mendelian disorders. J Allergy Clin Immunol. 2017;139(1):232-45.

2. Lundman E, Gaup HJ, Bakkeheim E, Olafsdottir EJ, Rootwelt T, Storrosten OT, et al. Implementation of newborn screening for cystic fibrosis in Norway. Results from the first three years. J Cyst Fibros. 2016;15(3):318-24.

3. Strand J, Gul KA, Erichsen HC, Lundman E, Berge MC, Tromborg AK, et al. Second-Tier Next Generation Sequencing Integrated in Nationwide Newborn Screening Provides Rapid Molecular Diagnostics of Severe Combined Immunodeficiency. Front Immunol. 2020;11:1417.

4. Hsu AP, Johnson KD, Falcone EL, Sanalkumar R, Sanchez L, Hickstein DD, et al. GATA2 haploinsufficiency caused by mutations in a conserved intronic element leads to MonoMAC syndrome. Blood. 2013;121(19):3830-7, s1-7.

5. Thorvaldsdóttir H, Robinson JT, Mesirov JP. Integrative Genomics Viewer (IGV): high-performance genomics data visualization and exploration. Briefings in bioinformatics. 2013;14(2):178-92.

6. Richards S, Aziz N, Bale S, Bick D, Das S, Gastier-Foster J, et al. Standards and guidelines for the interpretation of sequence variants: a joint consensus recommendation of the American College of Medical Genetics and Genomics and the Association for Molecular Pathology. Genet Med. 2015;17(5):405-23.
